# Short-term effect of low-dose roxadustat combined with erythropoiesis-stimulating agent treatment for erythropoietin-resistant anemia in patients undergoing maintenance hemodialysis

**DOI:** 10.3389/fendo.2024.1372150

**Published:** 2024-07-01

**Authors:** Qiaoying Xu, Jingjing Huang, Qingzhen Liu, Xueling Wang, Haiying Liu, Yan Song, Fulin Dou, Shasha Lv, Gang Liu

**Affiliations:** ^1^ Department of Nephrology, Multidisciplinary Innovation Center for Nephrology, The Second Hospital, Cheeloo College of Medicine, Shandong University, Jinan, China; ^2^ Nephrology Research Institute, Shandong University, Jinan, China; ^3^ Emergency Department, Caoxian People’s Hospital, Heze, China; ^4^ Key laboratory of Reproductive Endocrinology of Ministry of Education, Shandong University, Jinan, China

**Keywords:** roxadustat, RhuEpo, patients undergoing hemodialysis, renal anemia, ESA resistance

## Abstract

**Background:**

Erythropoietin resistance is present in some patients with chronic kidney disease, especially in those undergoing hemodialysis, and is often treated using roxadustat rather than iron supplements and erythropoiesis-stimulating agents (ESAs). However, some patients cannot afford full doses of roxadustat. This retrospective study investigated the efficacy of low-dose roxadustat combined with recombinant human erythropoietin (rhuEPO) therapy in 39 patients with erythropoietin-resistant renal anemia undergoing maintenance hemodialysis (3-4 sessions/week).

**Methods:**

The ability of the combination of low-dose roxadustat and rhuEPO to increase the hemoglobin concentration over 12 weeks was assessed. Markers of iron metabolism were evaluated. Eligible adults received 50–60% of the recommended dose of roxadustat and higher doses of rhuEPO.

**Results:**

The mean hemoglobin level increased from 77.67 ± 11.18 g/dL to 92.0 ± 8.35 g/dL after treatment, and the hemoglobin response rate increased to 72%. The mean hematocrit level significantly increased from 24.26 ± 3.99% to 30.04 ± 3.69%. The soluble transferrin receptor level increased (27.29 ± 13.60 mg/L to 38.09 ± 12.78 mg/L), while the total iron binding capacity (49.22 ± 11.29 mg/L to 43.91 ± 12.88 mg/L) and ferritin level (171.05 ± 54.75 ng/mL to 140.83 ± 42.03 ng/mL) decreased.

**Conclusion:**

Therefore, in patients with ESA-resistant anemia who are undergoing hemodialysis, the combination of low-dose roxadustat and rhuEPO effectively improves renal anemia and iron metabolism.

## 1 Introduction

The worldwide prevalence of chronic kidney disease (CKD) is increasing. In China, there are an estimated 120 million patients with CKD and approximately 2% will progress to end-stage renal disease (ESRD) (1, 2).

Renal anemia is a common complication of CKD, and its incidence increases gradually with the progression of CKD. More than 90% of patients with ESRD have renal anemia, which increases the risks of cardiovascular events and all-cause mortality and is associated with a poorer quality of life (3, 4). Erythropoiesis-stimulating agents (ESA) and iron supplements are typically used to treat anemia in patients with CKD and result in an increase of the hemoglobin (Hb) concentration to the goal of 10–11 g/dL in the USA and 10–12 g/dL in other parts of the world. However, 5–10% of patients with CKD and 10–15% of patients with CKD-5D (chronic kidney disease patients` stage 5,on regular HD) are hyporesponsive to ESAs and remain anemic despite treatment with high doses of ESA (4, 5). These patients also require frequent blood transfusions.

The factors contributing to ESA hyporesponsiveness in patients with CKD-5D include absolute or functional iron deficiency, acute or chronic inflammation (which may induce ESA hyporesponsiveness by increasing serum hepcidin,IL-6 and hsCRP levels), concurrent infections, nutritional deficiencies, insufficient dialysis, low albumin levels, muscle mass, and adipose tissue levels (5, 6). ESA hyporesponsiveness is associated with higher mortality rates and poorer clinical outcomes. The anemia must be corrected effectively, which often requires higher doses of rhuEPO, leading to a higher incidence of treatment-related adverse events, such as tumors, elevated blood pressure, endocrine system dysfunction, stroke, and cardiovascular events (2, 7–15). Although it remains unclear whether adverse effect prognoses are a result of the direct toxicity of ESA or of underlying conditions requiring high doses of ESA (15), we should limit the dosage to a suitable range, which can increase the HB concentration without increasing the risk of side effects. Roxadustat (FG-4592), a novel oral agent, has been incorporated into the therapeutic regimen for anemia in patients with CKD with or without hemodialysis (16–19). Roxadustat is a hypoxia-inducible factor prolyl hydroxylase inhibitor(HIF-PHI) that increases endogenous erythropoietin concentrations and improves iron absorption and utilization by mediating hypoxia-inducible factors(HIF) (2). Roxadustat can significantly improve the Hb concentration and reduce the need for intravenous iron supplementation and transfusion (20, 21). Roxadustat may be as effective or more effective than conventional ESA therapy in correcting the anemia in patients who are hyporesponsive to ESA (5, 21).

The proportion of patients with CKD-5D with ESA hyporesponsiveness is high, and ESA alone cannot maintain the Hb concentration within the target range. Increased ESA doses are limited by adverse effects, and roxadustat is expensive. Therefore, roxadustat and rhuEPO have been combined to treat patients with CKD-5D with renal anemia. This retrospective study investigated the efficacy of lower-dose roxadustat combined with rhuEPO for the treatment of anemia in patients with CKD-5D with ESA hyporesponsiveness.

## 2 Patients and methods

### 2.1 Patients

Patients with CKD-5D with renal anemia and ESA hyporesponsiveness treated at the hemodialysis center of the Second Hospital of Shandong University in China between January 2020 and December 2022 were included in this study. All patients were 18–88 years of age with stage 5 CKD who underwent hemodialysis 3–4 times per week for >12 weeks and were in a state of adequate dialysis. The adequacy of dialysis was assessed by the spKt/V(single-pool Kt/V), using the second-generation Daugirdas formula (Kt/V= -ln(R-0.008×t) + [(4 -3.5×R)×UF/W], where R= post-dialysis blood urea nitrogen (BUN)/pre-dialysis BUN; t = dialysis time(in hours);UF= net ultrafiltration (in liters) during the HD session; W = post-dialysis weight (in kilograms) (22). Adequate dialysis was defined as spKt/V≥1.2 for patients undergoing dialysis 3 times a week and spKt/V≥ 0.8 for patients undergoing dialysis 4 times a week based on a historical value obtained within the prior month (23).

**Figure 1:**
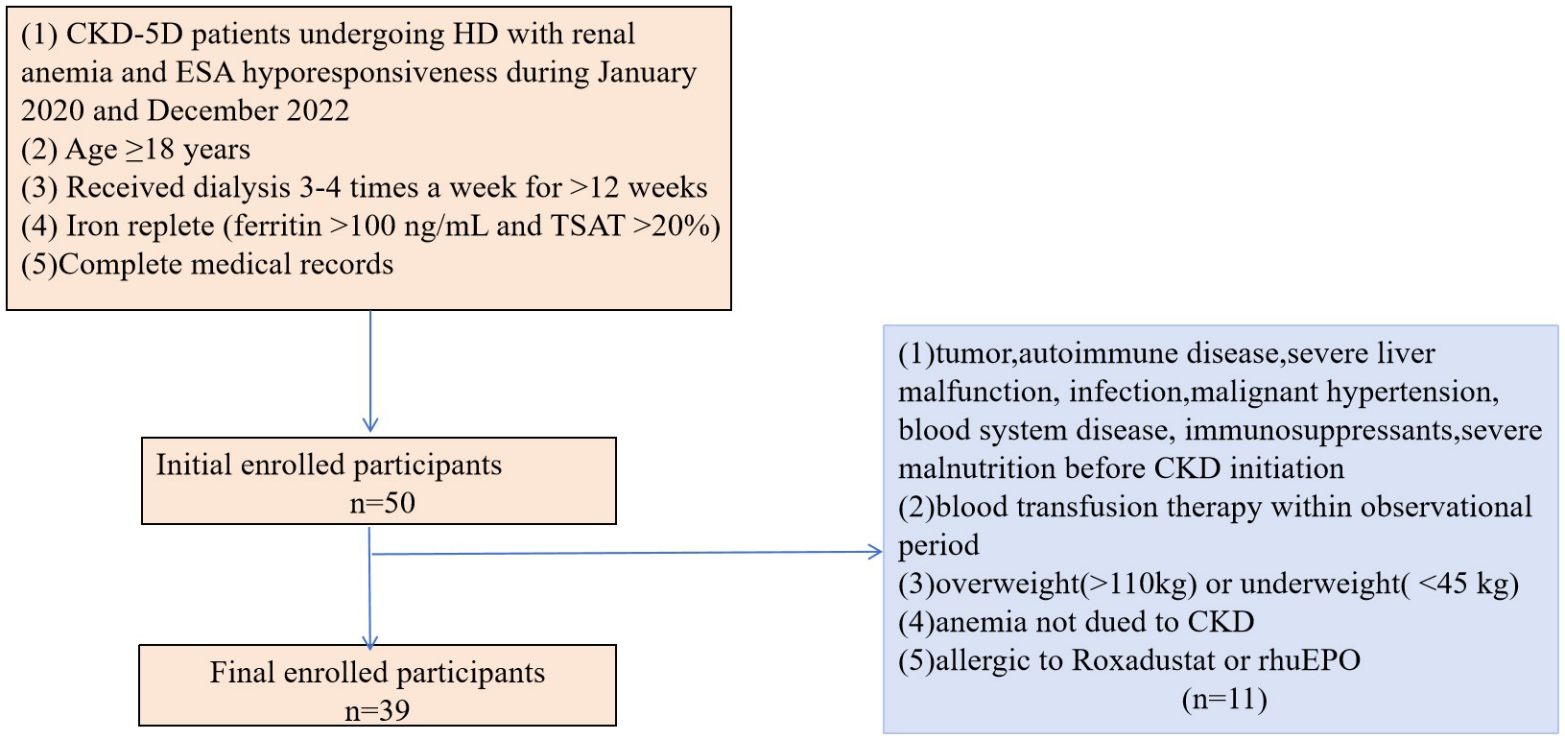
Participants inclusion chart.

In addition, all patients included in the study had anemia, defined as an Hb concentration between 60–110 g/L. The patients also had iron sufficiency at baseline (ferritin >100 ng/mL and transferrin saturation(TSAT) >20%) (5) and ESA resistance. ESA resistance was defined as a Hb concentration <11g/dL after at least three months of treatment with a dose of ESA(erythropoietin (>300 IU/kg/week or 20,000 IU/week) or darbepoetin-alfa (1.5 mg/kg or 100 mg/week)) or with a certain number of ESA resistance index(ERI) (≥1IU/Kg/wk/g/L for erythropoietin-treated patients and ≥0.005g/Kg/wk/g/L for darbepoetin-treated patients) or the requirement of a high dose of ESA to maintain the target range of Hb concentration. The ERI was defined as:ERI (IU/wk per kg Hgb [g/l] = (prior rhEPO dose [IU/kg per week])/(baseline Hgb [g/l]) (22, 23). The complete medical records were available for all included patients. Patients with tumors, autoimmune disease, severe liver malfunction, infection, malignant hypertension, blood system diseases, immunosuppressant use, or severe malnutrition prior to CKD were excluded from the study as were those who received a blood transfusion during the observation period. Patients with anemia not due to CKD, allergies to roxadustat or rhuEPO, or obesity (>110 kg) and those who were underweight (<45 kg) were also excluded from the study (**Figure 1**).

Written informed consent was obtained from each patient, and the study was approved by the Ethics Committee of the Second Hospital of Shandong University (approval number: KYLL-2023LW072). This study was conducted in accordance with the principles of the Declaration of Helsinki.

### 2.2 Study design

This retrospective, before-and-after control study was conducted for 12 weeks, during which all patients received rhuEPO and low-dose roxadustat. The roxadustat dose was 50–60% of the recommended dose in China (50 mg, three times per week for patients weighing 45–60 kg and 70 mg, three times per week for patients weighing ≥60 kg). No participants received iron supplementation during the study period.

Hb and hematocrit (HCT) data obtained at two, four, eight, and 12 weeks of treatment, and iron metabolism data obtained at four, eight, and 12 weeks of treatment were collected from the medical records. The Hb response rate was also determined. Hb response was defined as an increase in Hb ≥10 g/L when the baseline Hb was <95 g/L or an increase in Hb ≥5 g/L when the baseline Hb was between 95–100 g/L. In patients with a baseline Hb ≥100 g/L, a response was defined as maintenance of the Hb concentration within the target range of 110–120 g/L with no decrease >5 g/L (2, 23).

### 2.3 Data collection

The patient’s demographic data, including age, sex, height, weight, body mass index (BMI), and comorbidities (hypertension and diabetes mellitus) were extracted from the medical records, as were the primary kidney disease, anemia-related parameters (Hb and HCT) and iron-related parameters (serum iron, transferrin saturation (TSAT), ferritin, total iron-binding capacity (TIBC) levels, and soluble transferrin receptor (sTfR) levels). These biomedical parameters were measured using a biochemical automatic enzyme analyzer at the Clinical Laboratory of the Second Hospital of Shandong University.

### 2.4 Statistical analysis

Continuous variables are expressed as mean and standard deviation. Categorical variables are presented as numbers and percentages. A repeated-measures analysis of variance and Mauchly’s Test of Sphericity were performed to compare the data at each timepoint. All statistical analyses were conducted using IBM SPSS Statistics 23 software. Statistical significance was set at P<0.05.

## 3 Results

### 3.1 Demographic and clinical characteristics

A total of 39 patients were enrolled in this study. The mean patient age was 57.59 ± 15.58 years and 53.85% of patients were male (**Table 1**). The primary renal diseases of the patients were chronic glomerulonephritis (n=19), diabetic nephropathy (n=15), and hypertensive nephropathy (n=5). The baseline Hb concentration was 77.67 ± 11.18 g/L. The mean rhuEPO dose at baseline was 24769.23 ± 3081.88 IU per week. The erythropoietin resistance index (ERI)was 5.07 ± 1.01 IU/kg/wk Hb[g/L].

**Table 1:** Demographic and baseline data of the 39 participants.

Variables	Values
Age(year)	57.59±15.58
Gender (male%)	53.85
Height (m)	1.65±0.07
Weight (kg)	65.12±10.45
Body mass index (kg/m2)	23.74±3.11
The primary diseases	
Diabetes mellitus (%)	15(38.46%)
Hypertension(%)	5(12.82%)
Primary glomerulonephritis(%)	19(48.72)
IL-6(pg/mL)	8.18±3.34
BUN(mmol/L)	22.07±8.07
Scr(umol/L)	466.82±166.08
spKt/V (%)	1.36±0.12
Hgb ( g/dl)	77.67±11.18
Prior rhEPO dose, IU/w	24769.23±3081.88
ERI, IU/wk per kg Hgb[g/L]	5.07±1.01

IL-6, interleukin-6; BUN, blood urea nitrogen; Scr, Serum creatinine; spKt/V, single-pool Kt/V, quality of dialysis; Hgb, hemoglobin; rhEPO, recombinant human erythropoietin; ERI, erythropoietin resistance index.

### 3.2 Hb and HCT

**Figure 2:**
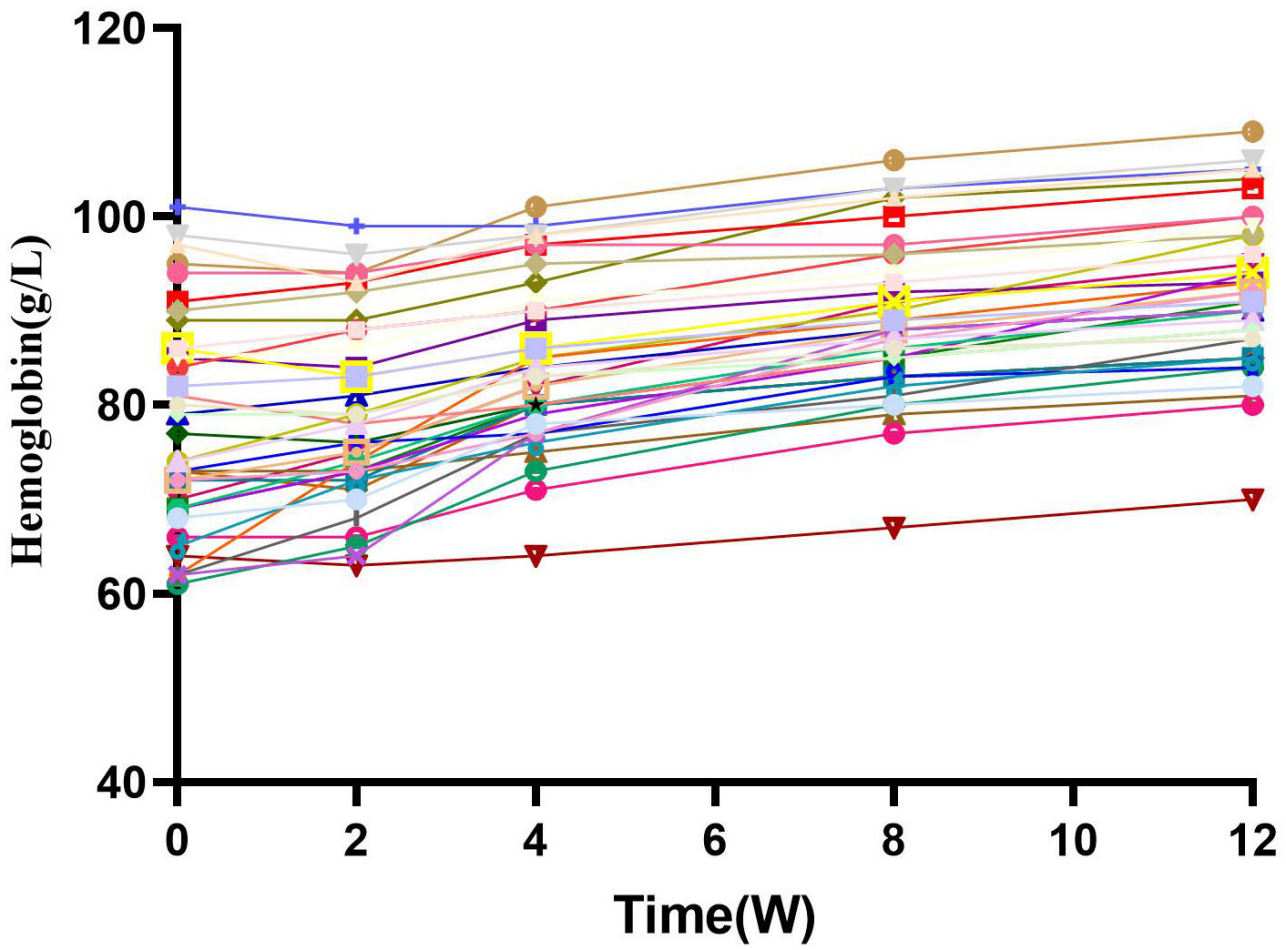
Hemoglobin and change from baseline over time. After combined treatment, HB levels gradually increased with time, and there was a significant statistical difference (P<0.05). W, Weeks.

The Hb concentration increased to 79.21 ± 9.63g/L after 2 weeks of treatment (**Figure 2**). The Hb concentration was 84.15 ± 8.58g/L after 4 weeks, 88.77 ± 8.27 g/L after 8 weeks, and 92.00 ± 8.35 g/L after 12 weeks (P< 0.01). The Hb response rate was 31% at 4 weeks and 72% at 12 weeks.

**Figure 3:**
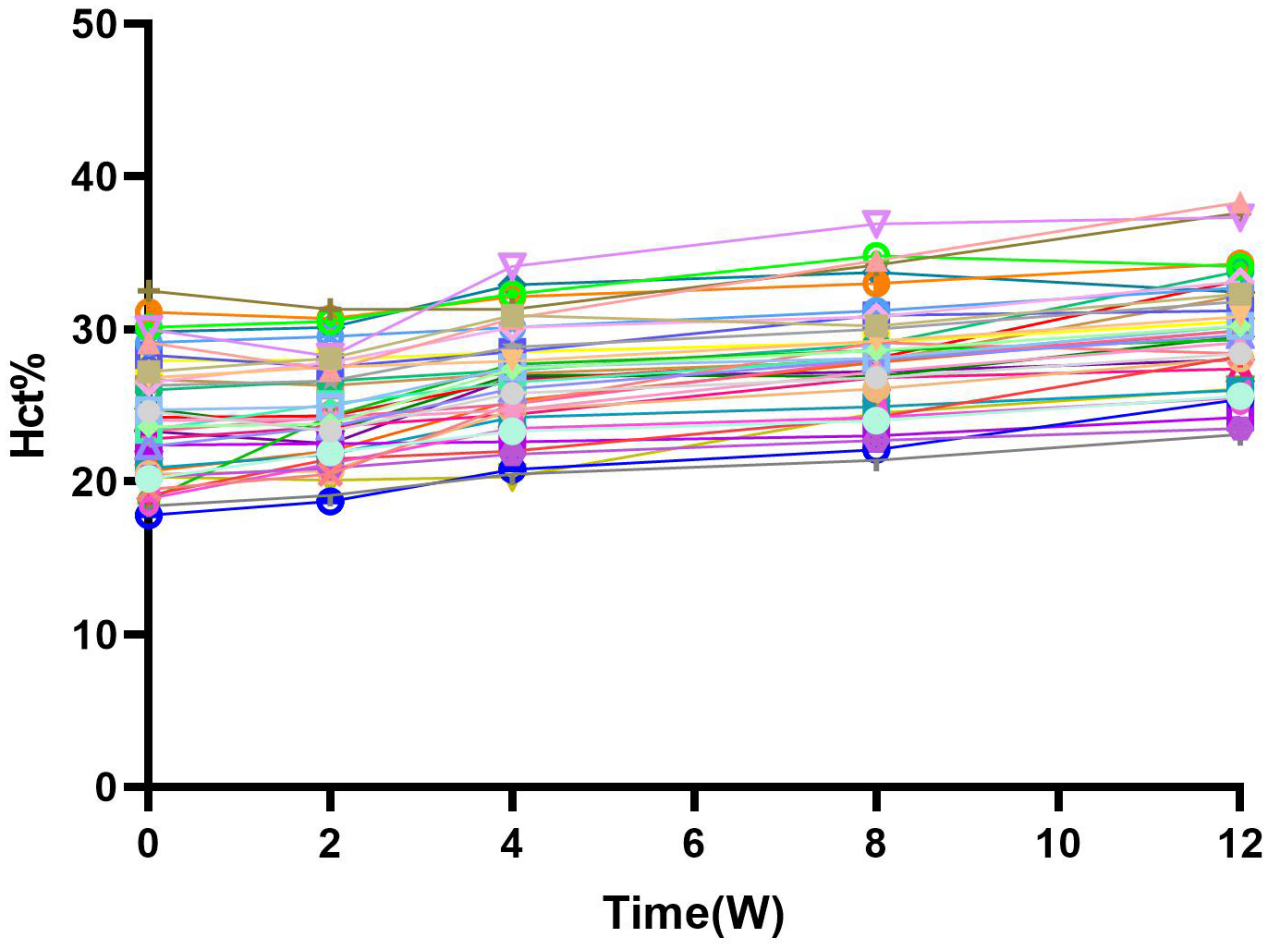
Hct and change from baseline over time. After the combination treatment, Hct level gradually increased over time, which corresponted with the change of HB, and there was a significant statistical difference(P<0.05). W, Weeks.

The HCT increased to 24.72 ± 3.41% at 2 weeks, 26.73 ± 3.53% at 4 weeks, 28.25 ± 3.66% at 8 weeks, and 30.04 ± 3.69% at 12 weeks (**Figure 3**). (p<0.01).

### 3.3 Effects of combination therapy on iron metabolism and utilization

The sTfR level significantly increased at each time point while the ferritin level and TIBC decreased continuously (**Table 2**). The TSAT was higher after 12 weeks (31.30 ± 7.25%) than at baseline (29.75 ± 8.07%), though the difference was not significant.

**Table 2:** Changes in iron−metabolism indices before and after combination therapy ($\bar{\text{X}}$±S).

Time	Ferritin (ng/mL)	SI (umol/L)	TIBC (umol/L)	TSAT (%)	sTfR (mg/L)
Baseline(Week 0)	171.05±54.75	14.36±4.58	49.22±11.29	29.75±8.07	27.29±13.60
Weeks 4	156.76±43.39	15.04±4.53	48.20±10.89	30.65±7.05	30.55±13.28
Weeks 12	140.83±42.03	14.12±4.40	43.91±12.88	31.30±7.25	38.09±12.78
F value	42.591	2.215	7.771	2.388	149.914
P value	<0.001	0.116	0.001	0.099	<0.001

After combination treatment, Ferritin and TIBC levels gradually decreased with statistical significance(P<0.05), while TSAT levels showed an upward trend, but there was no statistical difference(P>0.05).SI, serum iron; TIBC, total iron-binding capacity; TSAT, transferrin saturation; sTfR, soluble transferrin receptor.

### 3.4 Impact on costs

We compared the cost of low-dose roxadustat combined with rhuEPO and the recommended dose roxadustat, and found that the combined treatment could save nearly ¥(4749.90 ± 907.69) per year (**Table 3**).

**Table 3:** Cost of drugs to correct renal anaemia($\bar{\text{X}}$±S).

Treatment	Drug	Cost(¥)/Week	Cost difference(¥)/Week	Cost difference(¥)/Year
Low-dose roxadustat and rhuEPO	roxadustat	210.04±30.56	91.10±17.41	4749.90±907.69
	rhuEPO	59.56±17.41		
Recommended-dose roxadustat	roxadustat	360.70±30.56		

The table lists the cost of low-dose roxadustat combined with rhuEPO and the recommended -dose roxadustat, showing that the combination treatment has significantly lower costs than recommended-dose roxadustat alone.

## 4 Discussion

In our study, the chronic inflammation was indeed present in patients with chronic renal failure, accompanied by higher IL-6 levels, which can aggravate patients’ hyporesponsive to ESAs, but inflammation does not affect the efficacy of roxadustat in the treatment of anemia which consistent with previous studies (24, 25). And roxadustat may improve patients’ iron metabolism by reducing inflammation and down-regulating hepcidin levels (22, 26, 27). As an important factor regulating iron homeostasis, hepcidin prevents the absorption and entry into the blood of food-derived iron by binding to ferroportin in the epithelial cells of the duodenum, and it is produced and secreted by hepatocytes, while HIF-PHIs could suppress hepatic hepcidin expression by upregulating the concentration of HIF (28). In addition, hepcidin is affected by inflammation, iron status, and the oxygen concentration (27, 29). In this study, iron metabolism was significantly improved with the lower levels of Ferritin and TIBC and higher levels of TSAT after treatment with roxadustat, and HB levels were else higher, which was consistent with previous studies (22, 26). This means that with the improvement of iron metabolism, hepcidin levels will also decrease, and we will conduct further studies to verify this. In addition, the exclusion criteria included in this study were strict, and common factors affecting patients’ reactivity to ESA, such as infection and inadequate dialysis, were removed, so the study results were credible.

This is the first study to investigate the combination of low-dose roxadustat and rhuEPO in patients with CKD-5D with hyporesponsiveness to ESAs. This combination resulted in statistical increases in the Hb concentration. The mean Hb concentration significantly and continuously increased throughout the study period. In addition, the ferritin level and TIBC continuously decreased and the STfR level increased after treatment due to increased iron utilization to promote Hb production. However, the Hb concentration did not reach the target range in any patient in this study, which may have been due to the low Hb concentration at baseline, an insufficient treatment time, or the low dose of roxadustat.

These results are consistent with those of previous clinical studies regarding the efficacy of HIF-Prolyl Hydroxylase (HIF-PH) inhibitors in patients with anemia who are hyporesponsive to ESAs (30). One previous study reported that 40% of patients with CKD-5D and hyporesponsiveness to ESAs achieved or remained within the Hb concentration target range (10.5–11.0 g/dL) after 12 weeks of treatment with daprodustat (23). Another study reported that the Hb concentration and iron utilization increased and the PTH decreased in patients with CKD-5D with hyporesponsiveness to ESAs after 8 weeks of treatment with roxadustat. Roxadustat improved anemia in patients with CDK-5D without diabetes who were hyporesponsive to ESAs (30).

A dose-dependent relationship between roxadustat and Hb response was observed in this study, which is consistent with the results of previous studies (18, 31–33). A previous study reported dose-dependent increases in Hb concentrations in patients with CKD and renal anemia after 6 weeks of treatment (34). Another study reported that the Hb response rate and Hb concentration increased in a dose-dependent manner in patients with CKD undergoing hemodialysis and in those who did not require hemodialysis during a 4-week treatment period with roxadustat (35). Prof. Shanzhai Wei et al. found that compared with the low-dose roxadustat combined with rHuEPO, the HCT,HB,TSAT and SF levels of patients in the high-dose roxadustat combined with rHuEPO group were significantly higher after 12 weeks of treatment (26). In this study, the Hb concentrations increased significantly after 4 weeks of treatment, though the response time was relatively late, which may be due to the low dose of roxadustat; however, most patients ultimately responded well.

Future studies should examine the increases in Hb concentrations with different doses of roxadustat. However, higher doses of roxadustat are expensive and increase the risk of adverse events, such as hypertension, hyperkalemia, thromboembolism, malignancy, cardiovascular events, and death (30, 36–38).

The combination of low-dose roxadustat and rhuEPO resulted in a mean cost savings of ¥(4749.90 ± 907.69)per year based on the 43% decrease in the roxadustat dose. This cost reduction significantly decreases the economic burden on patients and may improve patient compliance.

In other words, low-dose roxadustat combined with erythropoietin therapy not only improves anemia and relieves ESA resistance but also reduces the risk associated with adverse events of higher doses of roxadustat, which is worthy of clinical reference and application in ESA-hyporesponsive patients.

However, this study is not without limitations. First, the retrospective design of this study introduces a patient selection bias. Interventional trials are needed to improve the generalizability of these data. Second, this study included a small patient population, limiting the opportunities for subgroup analyses of different dose regimens and different underlying diseases. Third, the study period was short, limiting the observation of adverse effects of roxadustat. More heterogeneous and long-term interventional studies should be performed to observe the effectiveness and safety of dosing adjustment strategies of combinations of roxadustat and ESAs for patients with CKD-5D and anemia who are hyporesponsive to ESAs.

In conclusion, low-dose roxadustat combined with rhuEPO improved anemia and iron metabolism in patients with CKD-5D and hyporesponsiveness to ESAs., which provides a new direction for the clinical treatment of this patient population. However, more studies with larger patient populations and a wider range of roxadustat doses are necessary.

## Data availability statement

The raw data supporting the conclusions of this article will be made available by the authors, without undue reservation.

## Ethics statement

The studies involving humans were approved by the Ethics Committee of the Second Hospital of Shandong University. The studies were conducted in accordance with the local legislation and institutional requirements. The participants provided their written informed consent to participate in this study. Written informed consent was obtained from the individual(s) for the publication of any potentially identifiable images or data included in this article.

## Author contributions

QX: Data curation, Methodology, Software, Writing – original draft, Writing – review & editing. JH: Data curation, Methodology, Writing – review & editing. QL: Writing – review & editing, Data curation, Methodology. XW: Data curation, Writing – review & editing. HL: Data curation, Writing – review & editing. YS: Data curation, Writing – review & editing. FD: Writing – review & editing, Data curation. SL: Writing – review & editing, Data curation. GL: Conceptualization, Funding acquisition, Methodology, Supervision, Writing – review & editing.
